# Impact of acute dynamic exercise on radial artery low-flow mediated constriction in humans

**DOI:** 10.1007/s00421-018-3876-1

**Published:** 2018-05-10

**Authors:** Robert O. Elliott, Sultan Alsalahi, James P. Fisher

**Affiliations:** School of Sport, Exercise and Rehabilitation Sciences, College of Life and Environmental Sciences, University of Birmingham, Edgbaston, Birmingham, UK

**Keywords:** Low-flow mediated constriction, Leg cycling, Vascular function

## Abstract

**Purpose:**

A “low-flow mediated constriction” (L-FMC) is evoked in the radial artery by the inflation of an ipsilateral wrist cuff to a supra-systolic pressure. We sought to test the hypothesis that the radial artery L-FMC response is augmented immediately following acute dynamic leg exercise in young healthy individuals.

**Methods:**

Ten healthy and recreationally active men (23 ± 4 years) undertook a 30-min trial of incremental dynamic leg cycling exercise (10 min at 50, 100 and 150 W) and a 30-min time control trial (seated rest). Trials were randomly assigned and performed on separate days. Radial artery characteristics (diameter, blood flow and shear rate) were assessed throughout each trial, with L-FMC and flow-mediated vasodilatation (FMD) assessments conducted prior to and immediately following (10 min) trials.

**Results:**

Dynamic leg cycling exercise increased radial artery blood flow, along with mean, retrograde and anterograde shear rate (*P* < 0.05). Blood flow profiles were unchanged during the time control trial (*P* > 0.05). Following exercise L-FMC was increased (mean [SD]; − 5.6 [3.3] vs. − 10.1 [3.8] %, *P* < 0.05), while it was not different in the time control condition (− 8.1 [3.2] vs. − 6.7 [3.4] %, *P* > 0.05). FMD was not different following either the exercise or time control trials (*P* > 0.05), but the composite end-point of L-FMC + FMD was enhanced post-exercise (*P* < 0.05).

**Conclusions:**

Dynamic exercise with a large muscle mass acutely augments the vasoconstrictor response of the radial artery in response to a reduction in blood flow (L-FMC) in young healthy individuals. The time course of this post-exercise response and the underlying vasoregulatory mechanisms require elucidation.

## Introduction

The endothelium fulfils a number of critical roles pertaining to the maintenance of vascular homeostasis (e.g., vasoregulation, hemostasis, and angiogenesis), while endothelial dysfunction is a hallmark of a plethora of pathophysiological conditions (Hambrecht et al. 2003; Maiorana et al. 2001; Watts et al. 2004). The “flow-mediated vasodilatation” (FMD) technique is a widely adopted non-invasive test of endothelial function (Corretti et al. 2002). In humans, this now typically involves the continuous monitoring of a conduit artery (e.g., brachial or radial arteries) using high resolution duplex Doppler ultrasound while a cuff placed distal to the measurement site is inflated for several minutes to occlude the downstream blood flow, before being rapidly deflated to induce a reactive hyperaemia and a distinct shear stress within the upstream artery. The magnitude of the ensuing vasodilatory response is prognostically significant in a number of populations (Ras et al. 2013), and the FMD mechanism is believed to be at least in part mediated by nitric oxide (Green et al. 2014; Joannides et al. 1995; Kooijman et al. 2008; Mullen et al. 2001). In contrast to the FMD response, it is much less well appreciated that the inflation of the cuff causing an occlusion during the FMD test produces a blood flow decrease through the upstream artery that induces a “low-flow mediated constriction” (L-FMC) (Gori et al. 2008; Humphreys et al. 2014; Levenson et al. 1987). L-FMC shows promise as clinically relevant biomarker of vascular function that provides complimentary information to that derived by the FMD technique (Gori et al. 2012, 2016). However, the underlying mechanisms and modulating factors (e.g., physical activity, environment, time of day) remain incompletely understood.

Exercise training is known to improve cardiovascular health (Fletcher et al. 2013) and to enhance peripheral vascular function (e.g., FMD) in some populations (Hambrecht et al. 2003; Watts et al. 2004). The repeated exercise-induced increases in arterial blood flow and shear stress appear to play an important role in this adaptation (Laughlin et al. 2008). However, immediately following an acute bout of dynamic exercise with a large muscle mass the brachial artery FMD response is reported to be attenuated, although this has not been a consistent observation and is modulated by exercise intensity, modality and duration (Dawson et al. 2013). While oxidative stress and hemodynamic factors may contribute to a post-exercising blunting of FMD, the heightened activation of the sympathetic nervous system is a prime candidate (Dawson et al. 2013). Following administration of an α_1_-adrenergic antagonist (prazosin), rather than there being a post-exercise attenuation, brachial artery FMD is actually augmented (Atkinson et al. 2015). The blunting of the post-exercise brachial FMD response may be a direct consequence of an increase in sympathetic vasoconstrictor tone and/or secondary to a sympathetically mediated increase in retrograde shear (Atkinson et al. 2015; Thijssen et al. 2014).

In contrast to FMD, the effect of acute dynamic exercise on L-FMC has not been examined. FMD and L-FMC responses are not well correlated suggesting that they occur by different mechanisms (Gori et al. 2008), therefore, in the post-exercise period L-FMC responses cannot be assumed to change in parallel with FMD. There is evidence that the L-FMC response is partly endothelial dependent (Dawson et al. 2012) and it is attenuated by the removal of certain vasodilatory substances (e.g., endothelial derived hyperpolarizing factors, prostaglandins) (Gori et al. 2008). However, is it neither affected by infusion of the nitric oxide synthase inhibitor blocker NG-monomethyl-l-arginine (Gori et al. 2008), nor does the magnitude of L-FMC correlate with the reduction in shear rate during cuff occlusion (Weissgerber et al. 2010). L-FMC is also diminished by the endothelin receptor (ET_A_) antagonist BQ-123 (Spieker et al. 2003), and a role for the sympathetic nervous system has been suggested, but not directly tested (Gori et al. 2008). Therefore, in the immediate post-exercise period an alteration in either vasodilatory or vasoconstrictor signalling pathways, may transiently increase the magnitude of the L-FMC response. However, the impact of acute dynamic leg exercise on radial artery low-flow mediated constriction, along with radial artery blood flow, patterns of shear rate and FMD, remain to be explored.

The present study was conducted in light of the importance of endothelial function to cardiovascular health, the paucity of information regarding the utility of L-FMC in evaluating vascular function, and the known effects of acute exercise on vasoregulation. We tested the hypothesis that acute dynamic leg exercise augments the radial artery L-FMC response in young healthy individuals.

## Methods

### Ethical approval

This study (ERN_17-0004) was approved by the University of Birmingham, Science Technology Engineering and Mathematics ethical review committee and undertaken in accordance with the most recent revision of the Declaration of Helsinki. Written informed consent was obtained from all participants after they had received a detailed verbal and written explanation of the study procedures.

### Participants

10 healthy men (age: 23 ± 4 years; height: 179 ± 6 cm; weight: 76.8 ± 6.8 kg) that were normotensive, non-smokers and medication free were recruited and recreationally active. Participants were recreationally active and engaging in aerobic activities for > 30 min on ≥ 3 days per week (e.g., swimming, walking, cycling). Prior to experimental sessions participants were requested to adhere to the following directions: no food or beverages ≥ 6 h, no alcohol or caffeine for ≥ 12 h, no polyphenol rich food/beverages for ≥ 18 h, no vigorous exercise for ≥ 48 h and no vitamin supplements for ≥ 72 h.

### Experimental measurements

Heart rate (HR) was monitored using a standard lead II surface electrocardiogram, while systolic and diastolic blood pressure (BP) were measured non-invasively from the left brachial artery using an automated sphygmomanometer (Tango+, SunTech Medical Instruments, Raleigh, NC, USA). Radial artery blood flow velocity and diameter were obtained from the right arm positioned at heart level by Doppler ultrasound (Logiq E; GE Medical Systems, Milwaukee, WI, USA) by a single experimenter (ROE), using a 10-MHz multi-frequency linear-array transducer. The probe was attached to an adjustable holder to obtain a stable longitudinal image of the radial artery 10–15 cm proximal to a narrow inflatable cuff placed around the right wrist (5 cm width, Hokanson, Bellevue, WA, USA). B-mode imaging was used to measure arterial diameter, and peak blood velocity was simultaneously measured using the pulse-wave mode. Measurements were made in accordance with recent technical recommendations (Thijssen et al. 2011). Radial artery recordings were screen captured and stored as video files for offline analysis (FMD Studio, Pisa, Italy). This software utilised image based automated edge detection and wall tracking algorithms working independently of investigator influence (Gemignani et al. 2007). All video files were analysed by a single operator (ROE) whom was blinded to the experimental conditions.

### Experimental design

Participants attended a familiarisation session prior to undertaking the experimental protocol, during which all the study procedures were conducted. Participants then returned to the laboratory on two separate occasions at the same time (between 9 am and 1 pm) and separated by at least 24 h, but ≤ 7 days. Laboratory temperature was maintained at ~ 22 °C. Upon arrival at the laboratory participants were seated and instrumented for the measurement of the recorded physiological variables described above. A 20-min rest period was undertaken, BP and HR were then measured after which radial artery endothelial function was assessed (i.e., L-FMC, FMD) from the right arm. This involved the monitoring of the radial artery velocity and diameter, before (2 min), during (5 min) and after (3 min) the inflation of a cuff placed around the right wrist to a supra-systolic pressure of 240 mmHg.

Participants then either rested for a further 30 min (time control trial) or undertook a 30-min bout of leg cycling (exercise trial). Trial order was randomly assigned with a coin toss and resulted in a similar number of participants undertaking the time control (*n* = 4) and exercise (*n* = 6) trials first. During cycling exercise, the workload was initially set at 50 W and then increased incrementally by 50 W at 10 min intervals, whilst cadence was maintained at 70 rpm. Upon completion of both the time control and cycling exercise trials a further 10-min recovery period was conducted. Radial artery blood flow velocity and diameter were measured for 60 s every 5 min throughout this 40-min period (i.e., exercise and recovery, or time control and recovery). Accompanying ratings of perceived exertion (RPE) (6–20 point Borg Scale) were obtained, along with HR and BP. Immediately after this 40-min period another assessment of radial artery endothelial function was undertaken (L-FMC, FMD). The right arm was maintained in the same position throughout all phases of the time control and exercise trials. Steady-state incremental exercise was used because it was believed to be tolerable by the participants and permitted high quality images to be obtained from the radial artery. This exercise modality (cycling) and workloads are relevant because they involve dynamic exercise of a large muscle mass at an intensity recommended for developing and maintaining cardiorespiratory fitness.

### Data analysis

Mean arterial pressure (MAP) was calculated as: diastolic BP + [0.33 + (HR × 0.0012)] × [systolic BP − diastolic BP] (Razminia et al. 2004). Steady-state measurements of systolic BP, diastolic BP, MAP and HR were obtained at baseline and every 5 min throughout the time control and cycling exercise trials. Radial artery blood flow was calculated using measurements of real time diameter and velocity using the following formula:

$${\text{Radial}}\;{\text{artery}}\;{\text{blood}}\;{\text{flow}}=\frac{{{\text{Peak}}\;{\text{envelope}}\;{\text{velocity~}}}}{2} \cdot [\pi ~{(0.5~ \cdot {\text{Diameter}})^2}]$$

Radial arterial vascular conductance was calculated as: radial artery blood flow/MAP. Shear rate was calculated as radial artery blood velocity multiplied by 4 and divided by radial artery diameter. Steady-state measurements of radial artery blood velocity, diameter and shear rate were obtained at baseline and every 5 min throughout the time control and cycling exercise trials. A minimum of 10 consecutive cardiac cycles were used in each experimental phase. In one participant, high quality radial artery signals were not obtainable during the final measurement point of leg cycling exercise (30 min). To maintain statistical power this single missing value in the radial artery exercise data was replaced with the mean of the respective group at that time point (Donders et al. 2006).

Throughout each radial artery endothelial function test of L-FMC and FMD, radial artery velocity, diameter, flow and shear rate were continuously monitored. Baseline values were first established as an average over the 2 min prior to wrist cuff inflation. The L-FMC response was taken as the radial artery diameter change from baseline to the average values calculated over the last 30 s of cuff occlusion (Gori et al. 2008). FMD was taken as the maximal increase in radial artery diameter change following cuff deflation. The time to the peak diameter was also obtained, along with the shear rate area under the curve (SR_AUC_) calculated between the cuff deflation and the maximal dilation. FMD and L-FMC responses are expressed in both relative (%) and absolute (mm) terms (Thijssen et al. 2011). Baseline radial artery diameter measurements showed a high within-day (i.e., time control pre vs. post-trials) and the between-day (i.e., time control pre vs. exercise pre trials) test–retest reliability. Specifically, the within-day measures had a Pearson correlation of 0.949 *P* > 0.001, an intraclass correlation of 0.956 (95% CI 0.752–0.990) *P* < 0.001 and a co-efficient of variation of 3%, whereas the between-day measures had a Pearson correlation of 0.638 *P* = 0.047, an intraclass correlation of 0.790 (95% CI 0.109–0.949), *P* = 0.019 and a co-efficient of variation of 5%.

### Statistical analysis

All statistical analysis was undertaken using SigmaPlot (version 13, Systat Software Inc). Normal distribution was evaluated using Shapiro–Wilk tests. Two-way repeated measures analysis of variance (ANOVA) was used to test for differences in systolic BP, diastolic BP, MAP, HR, radial artery velocity, diameter, flow and shear rate with respects to time (BL, 5, 10 15, 20 and 30 min) and condition (time control and exercise). A two-way repeated measures ANOVA was also used to test for differences in time (pre, post) and trial (rest, exercise) for baseline diameter, L-FMC, FMD and SR_AUC_. Friedman repeated measures ANOVA on ranks was used to test for differences in RPE during exercise. Post hoc analysis was undertaken using the Student–Newman–Kuels method to investigate significant main effects and interactions. *P* < 0.05 was considered as being statistically significant. Data are presented as mean (SD) unless otherwise specified.

## Results

### Arterial pressure and heart rate

**Fig. 1:**
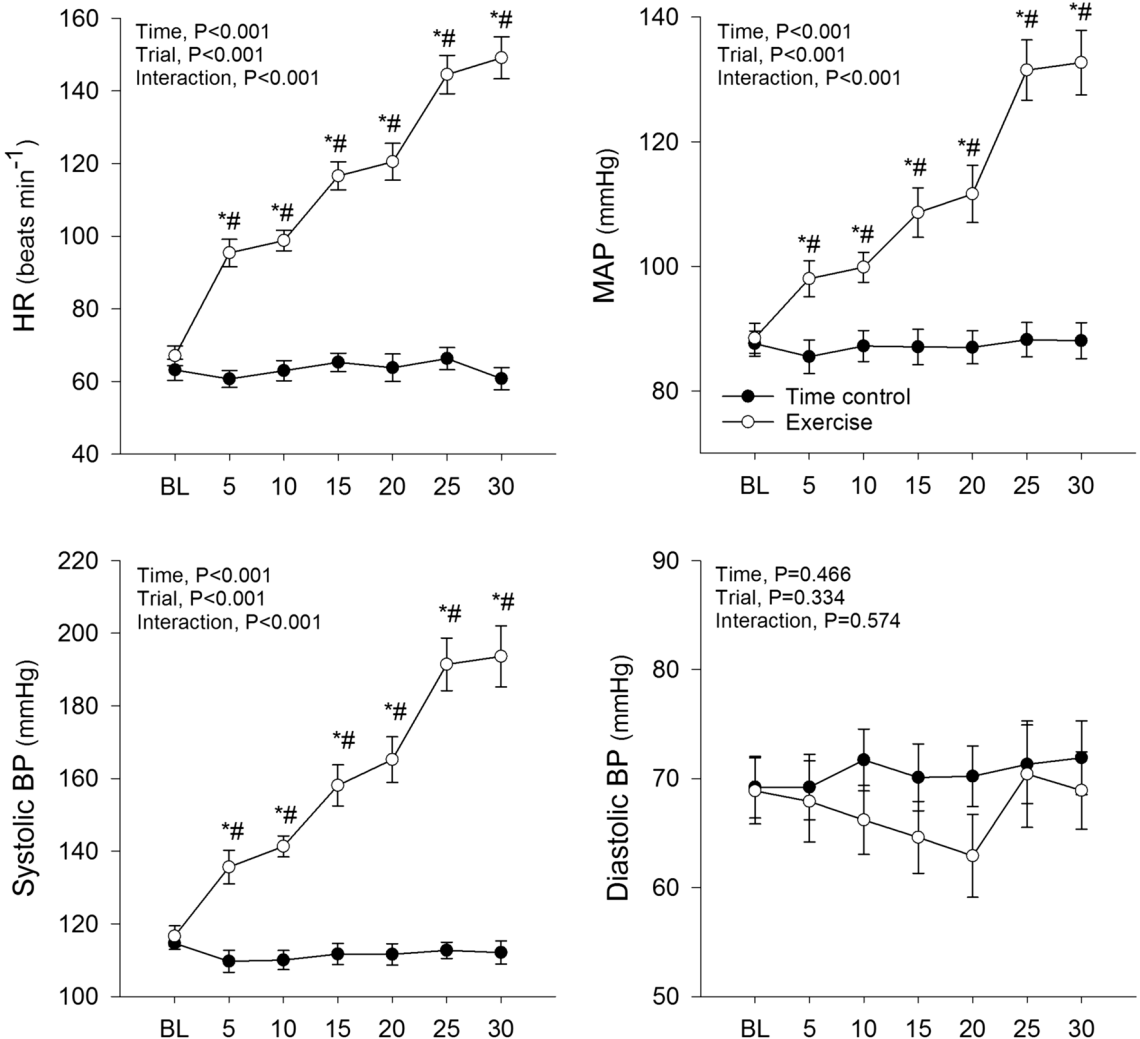
Heart rate (HR), mean arterial pressure (MAP), systolic blood pressure (systolic BP) and diastolic blood pressure (diastolic BP) during time control and leg cycling exercise trials. The exercise protocol consisted of three 10-min bouts of leg cycling conducted consecutively at 50, 100 and 150 W. Values are mean ± SE. *P < 0.05 vs. baseline (BL); ^#^*P* < 0.05 vs. time control trial

HR, systolic BP and MAP increased significantly from baseline during leg cycling exercise (*P* < 0.05), but remained unchanged during the time control trial (Fig. 1). Diastolic BP was not significantly changed from baseline in either the time control or exercise trials. RPE increased during exercise along with each increment in workload (9 [7–9] (median [interquartile range]), 9 [8–11], 12 [11–12], 13 [11–13], 15 [14–15] and 16 [15–17] at 5, 10, 15, 20, 25 and 30 min time points, respectively; *P* < 0.05).

### Blood flow and shear rate

**Fig. 2:**
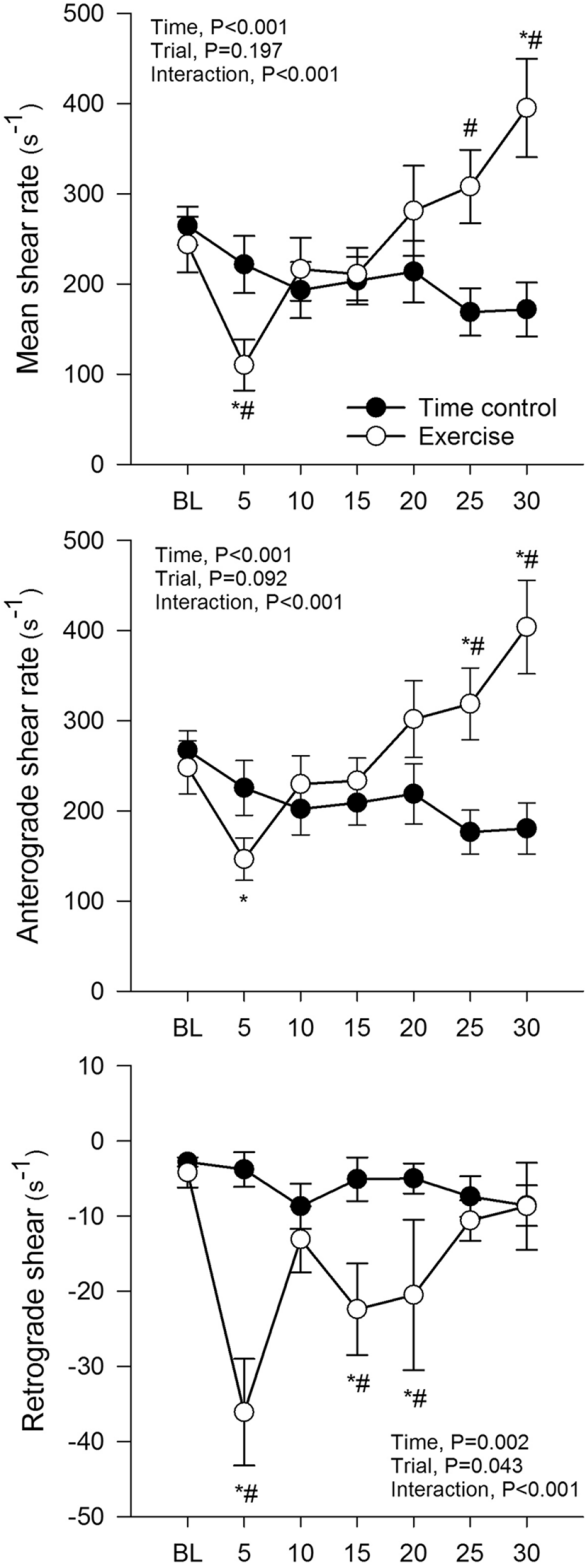
Mean, anterograde and retrograde shear rate in the radial artery during time control and leg cycling exercise trials. Values are mean ± SE. *P < 0.05 vs. baseline (BL); ^#^*P* < 0.05 vs. time control trial

**Fig. 3:**
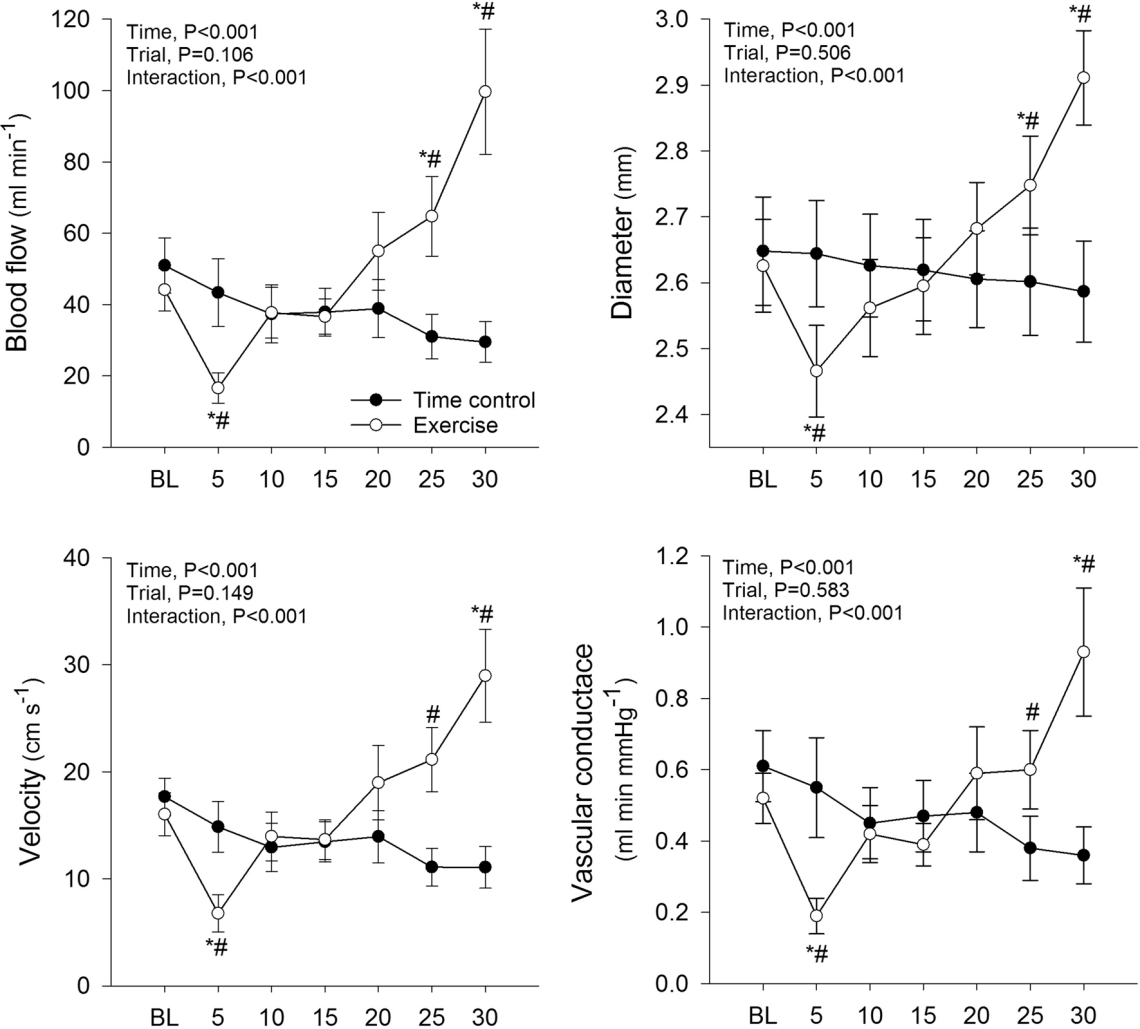
Radial artery blood flow, diameter, velocity and vascular conductance during time control and leg cycling exercise trials. Values are mean ± SE. *P < 0.05 vs. baseline (BL); ^#^*P* < 0.05 vs. time control trial

Baseline mean, anterograde and retrograde shear rate were not different between conditions (*P* > 0.05; Fig. 2). Mean and anterograde shear rate initially decreased from baseline during the first 5 min of exercise (*P* < 0.05), and then progressively increased such that they were significantly elevated above the time control condition at the 25 and 30 min time points. Retrograde shear rate was significantly increased at the start of the first exercise workload (baseline vs. 5 min, *P* < 0.05) and remain elevated during the second workload (i.e., 15 and 20 min), before returning to baseline levels during the final exercise workload (i.e., 25 and 30 min). Mean, anterograde and retrograde shear rate were unchanged from baseline in the time control condition. Radial artery blood flow, diameter, velocity and vascular conductance were not different between conditions at baseline (Fig. 3). All fell transiently at the start of exercise (baseline vs. 5 min, *P* < 0.05), before recovering to baseline and then increasing such that they were elevated above the time control condition at the 25 and 30 min time points (*P* < 0.05). Radial artery blood flow, diameter, velocity and vascular conductance were unchanged from baseline in the time control condition.

### L-FMC and FMD

**Fig. 4:**
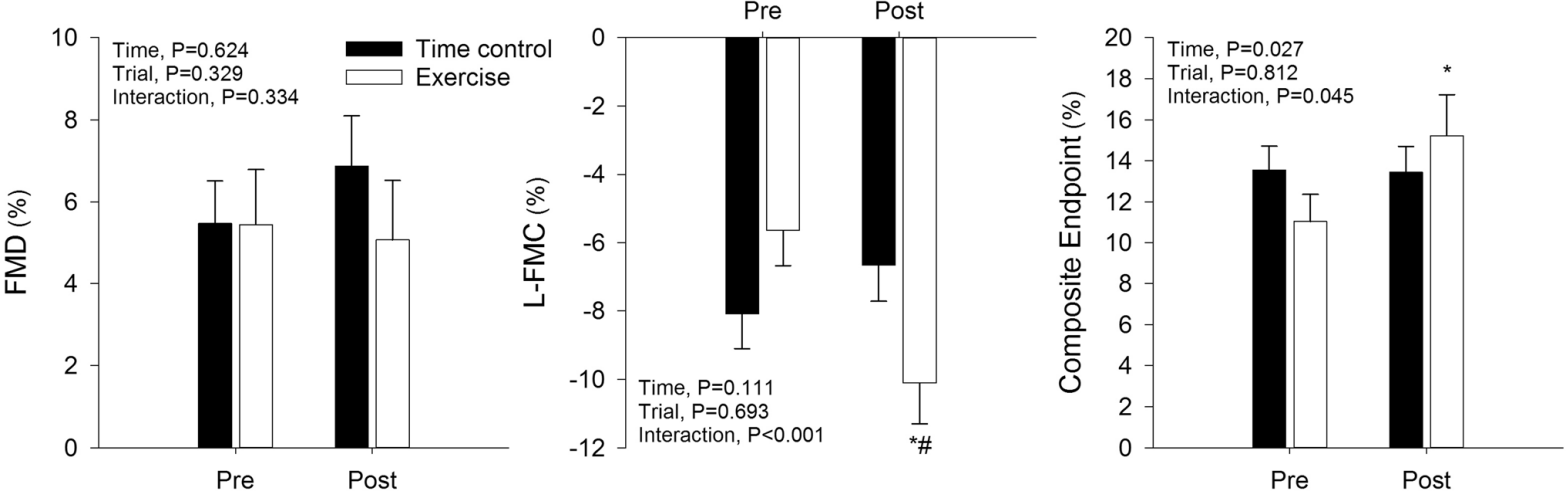
Radial artery low-flow mediated constriction (L-FMC), flow mediated dilatation (FMD) and the composite end point of L-FMC + FMD before (pre) and after (post) a time control period and leg cycling exercise. Values are mean ± SE. *P < 0.05 vs. baseline (BL); ^#^*P* < 0.05 vs. time control trial

Radial artery characteristics before (pre) and after (post) the time control and leg cycling exercise trials are provided in Table 1 and Fig. 4. L-FMC was significantly increased following exercise (pre vs. post, *P* < 0.05; time control vs. exercise, *P* < 0.05), while it was not significantly different in the time control condition. This was the case irrespective of whether L-FMC was expressed as the percentage change or absolute (mm) change in diameter. FMD and SR_AUC_ were not significantly different in the time control or exercise trials. The time to peak diameter was significantly decreased following exercise (pre vs. post, *P* < 0.05; time control vs. exercise, *P* < 0.05).

**Table 1:** Radial artery characteristics before (pre) and after (post) a time control period and leg cycling exercise

	Time control		Exercise		*P* values		
	Pre	Post	Pre	Post	Trial	Time	Interaction
Baseline							
Diameter (mm)	2.65 (0.26)	2.58 (0.30)*	2.63 (0.23)	2.74 (0.25)*^,#^	0.288	0.186	0.002
Velocity (cm s^−1^)	17.7 (5.5)	9.8 (5.2)*	16.0 (6.3)	19.3 (6.2)^#^	0.008	0.091	0.002
Blood flow (ml min^−1^)	50.9 (24.6)	27.0 (18.6)*	44.2 (18.7)	58.3 (22.7)*^,#^	0.006	0.234	0.002
Mean shear rate (s^−1^)	264.5 (21.3)	151.3 (71.7)*	244.0 (97.6)	282.4 (86.0)^#^	0.023	0.055	0.003
L-FMC							
Diameter (mm)	2.44 (0.26)	2.41 (0.30)	2.48 (0.23)	2.47 (0.26)	0.542	0.496	0.637
Δ diameter (mm)	− 0.22 (0.09)	− 0.17 (0.08)	− 0.15 (0.08)	− 0.28 (0.10)*^,#^	0.527	0.096	0.003
Mean shear rate (s^−1^)	41.3 (27.9)	41.6 (32.0)	47.4 (21.0)	28.3 (35.4)	0.676	0.387	0.304
FMD							
Peak diameter (mm)	2.79 (0.28)	2.76 (0.38)	2.76 (0.21)	2.88 (0.23)*	0.458	0.168	0.013
Δ Diameter (mm)	0.14 (0.09)	0.18 (0.11)	0.14 (0.11)	0.13 (0.12)	0.306	0.590	0.370
Time to peak diameter (s)	114.7 (17.5)	119.5 (20.2)	119 (12.9)	97.4 (21.6)*^,#^	0.062	0.044	0.004
SR_AUC_ (× 10^3^ s^−1^)	22.4 (9.6)	22.1 (7.7)	17.32 (6.1)	20.7 (9.8)	0.118	0.524	0.364

Values are means ± SD

*L-FMC* low-flow mediated constriction, *FMD* flow mediated dilatation, *SRAUC* shear rate area under curve. *P values P* values represent two-way repeated ANOVA results (trial, time control and exercise; time, pre and post; interaction, trial × time)

*P < 0.05 vs. pre; ^#^*P* < 0.05 vs. time control

## Conclusions

The major novel finding of the present study is that acute dynamic exercise with a large muscle mass augments the radial artery L-FMC response in young healthy individuals. In contrast, we did not observe an increase in the FMD response, whereas the composite end point (i.e., L-FMC + FMD) was augmented secondary to the greater L-FMC response. A context to these findings will be provided with respect to the relevant literature and experimental limitations in the following discussion.

In the mid-1980s, duplex Doppler ultrasound studies of brachial artery diameter provided in vivo evidence in humans that an acute reduction in blood flow induced by the inflation of a proximal cuff inflated to a supra-systolic pressure evoked a vasoconstriction in the upstream vessel (Anderson and Mark 1989; Levenson et al. 1987). The magnitude of this so-called L-FMC response relates to the reductions in local blood flow and, therefore, quantifies the vascular response to prevailing levels of shear stress or other factors implicated in establishing basal vascular tone (Humphreys et al. 2014). L-FMC, therefore, provides complimentary information to that derived by the FMD technique, which assesses the endothelium’s capacity to synthesise and release vasodilatory mediators (Thijssen et al. 2011). While FMD has been shown to have prognostic value in several populations (Ras et al. 2013), the clinical utility of L-FMC has been less well explored, but it is reported to be blunted in several patient populations (e.g., hypertension, coronary artery disease) (Gori et al. 2008), and when used alongside FMD the ability to discriminate between healthy individuals and those with cardiovascular disease is improved (Gori et al. 2010).

The mechanisms regulating blood vessel diameter are complex and multifactorial, and there are several potential explanations for the enhanced radial artery L-FMC response we observed following dynamic leg exercise. An enhancement in vasoconstrictor pathways may account for the observed post-exercise increase in L-FMC. L-FMC is known to be diminished by the ET_A_ receptor antagonist BQ-123 (Spieker et al. 2003) and a role for oxidative stress and the sympathetic nervous system has been implied (Gori et al. 2008; Humphreys et al. 2014), thus with the induction of a low-flow state a more robust vasoconstrictor response may be exhibited if these pathways are upregulated in the post-exercise period (Ichinose et al. 2008; Maeda et al. 1997). Heightened sympathetic nerve activity has previously been demonstrated to account for the reduction in brachial FMD immediately following exercise (Atkinson et al. 2015), although it should be recognised that FMD and L-FMC responses are not well correlated and likely occur by different mechanisms (Gori et al. 2008), so whether the sympathetic nervous system explains the greater post-exercise L-FMC requires investigation.

A decreased basal radial artery tone in the post-exercise period could also account for a more marked vasoconstriction being evoked when it is experimentally placed in a low-flow state. Radial artery diameter prior to assessment of L-FMC was observed to be enhanced post-exercise and may be attributable to both endothelial dependent and independent mechanisms. Dawson and colleagues provided evidence for the important role of the endothelium to the L-FMC response with the observation that endothelial damage induced by radial artery catheterization significantly blunts the L-FMC response in that vessel (Dawson et al. 2012). During the first two workloads of our incremental dynamic leg exercise protocol an increase in radial artery retrograde shear rate was observed, which has previously been shown to induce endothelial dysfunction (Thijssen et al. 2014). However, radial FMD was unchanged in the after-exercise period in the present study, suggesting that endothelial function was not changed. While a role for nitric oxide in the L-FMC response is disputed, L-FMC is attenuated by the removal of certain vasodilatory substances (e.g., endothelial derived hyperpolarizing factors, prostaglandins) (Gori et al. 2008) and an upregulation of these pathways may have contributed to the enhanced L-FMC response observed following exercise. The larger radial artery diameter noted post-exercise can complicate the interpretation of L-FMC when it is solely expressed as a percentage change. However, we observed that the L-FMC response in the radial artery was enhanced post-exercise irrespective of whether the change was expressed in absolute units (i.e., mm) or as a percentage, suggesting that this response was not an artefact attributable to a change in baseline.

We elected to examine the L-FMC responses to low-to-moderate dynamic leg cycling exercise as such an exercise mode and intensity is recommended for the enhancement and maintenance of cardiorespiratory fitness in diverse populations (Fletcher et al. 2013). In line with our observations, Gori et al. (2010) observed that 4 min of isometric handgrip (of unspecified intensity), enhanced radial artery L-FMC and attenuated the FMD. Our findings in part support these valuable observations, and extend them on account of several notable differences between the experimental paradigm employed. The mode of exercise (isometric vs. dynamic), the mass of the exercising muscle (small vs. large), the duration of exercise (4 vs. 30 min), and the proximity of the artery under examination to the exercising muscle groups (local vs. systemic effects) all have vasoregulatory implications. Observations based on the results of isometric exercise cannot be assumed to represent those manifested following dynamic exercise, due to differences in the hemodynamic and neural responses (Fisher et al. 2015). Whereas during low-to-moderate intensity dynamic exercise, skeletal muscle blood flow increases and limits the magnitude of metabolite accumulation in the contracting muscles during isometric exercise the mechanical compression of the contracting muscles can restrict muscle blood flow (Barcroft and Millen 1939). The ensuing mismatch between oxygen demand and delivery leads to metabolite accumulation and activation of the muscle metaboreflex, which evokes a powerful sympatho-excitation, vasoconstriction and a marked pressor response (Fisher et al. 2015). We observed that during dynamic leg cycling exercise radial artery blood flow increased in an intensity dependent manner, and would expect that during isometric handgrip increases in radial artery blood flow would be comparatively modest and inversely related to the force of contraction (Thompson et al. 2007). These factors all have implications for the ensuing L-FMC and FMD responses.

Following isometric handgrip exercise, the radial artery composite end-point (i.e., L-FMC + FMD) is unchanged (Gori et al. 2010), as the enhanced L-FMC is accompanied by a decreased FMD. In contrast, immediately following dynamic leg cycling exercise we observed that the composite end-point was enhanced. The advantage of assessing the composite end-point is that it provides an evaluation of the entire vasoactive range. The present investigation is the first to demonstrate that the vasoactive range of the radial artery is enhanced in the period immediately following dynamic exercise.

There are several experimental considerations that should be taken into account when interpreting the present results. Vascular function was only evaluated at a single time point following exercise. Some studies have shown that the FMD response in the post-exercise period is time dependent (Birk et al. 2013), and a similar appraisal of the time course of the L-FMC response to acute exercise is required. Vascular assessments were only made in the radial artery, and it is unclear whether the results of the present study are generalizable to other arteries. Although investigations have reported L-FMC in the brachial artery (Rakobowchuk et al. 2012) it is more commonly assessed in the radial artery, likely due in part to the observations of Weissgerber et al. (2010) who reported that L-FMC was more readily observed in the radial than the brachial artery. The radial artery characteristics (diameter, blood flow and shear rate) we observed during leg cycling exercise are similar to that previously documented for the brachial artery (Padilla et al. 2011; Simmons et al. 2011). At the onset of exercise (i.e., first 5 min) radial artery diameter, velocity and blood flow were decreased, after which an intensity dependent increase in these variables was observed. This initial vasoconstriction may relate to cutaneous and skeletal muscle vasoconstriction in the forearm and hand associated with the redistribution of blood flow to the exercising skeletal muscle (Johnson 2010), and/or reductions in mean and anterograde shear rate along with an increase in retrograde shear (Padilla et al. 2011). Cutaneous vasoconstriction may also explain the unexpected reduction in radial artery characteristics in the observed pre vs. post the time control condition, although the laboratory was maintained at constant thermoneutral temperature. Irrespective of these small changes in diameter L-FMC was not different in the pre vs. post time control condition, which concurs with Gori et al. (2010) who demonstrated that baseline diameter is not significantly correlated with L-FMC under resting conditions. We acknowledge that only young healthy men participated in this study and, therefore, caution should be exercised when generalising our findings to other groups, particularly populations in whom disruptions in vasodilatory and vasoconstrictor signalling have been identified (e.g., hypertension, coronary artery disease). In contrast to some studies (Dawson et al. 2013), we observed FMD to be unchanged from baseline in the post-exercise period. This attenuated response is observed to be less evident in trained individuals, and following exercise of a lower intensity (e.g., < 80% *V*O_2max_), and principally the brachial artery has been examined. Therefore, the aerobic condition of the participants recruited, the use of an incremental exercise protocol with a limited high-intensity component, and the examination of the radial artery may all have contributed to our findings.

Both exercise and the recovery period from exercise are times of increased cardiovascular risk (Albert et al. 2000; Mittleman and Siscovick 1996; Siscovick et al. 1984), with some abnormalities occurring specifically in the post-exercise period (Akutsu et al. 2002). Parallels exist between the vasodilatory behaviour of the peripheral and coronary vasculature (Anderson et al. 1995; Takase et al. 1998); however, whether L-FMC responses are representative of the coronary circulation requires further study. If the coronary blood vessels respond in a similar way to the radial artery, and exhibit an enhanced vasoconstrictor response during the immediate post-exercise period, this may in part contribute to increased cardiovascular risk experienced by some individuals at this time. In addition to these potential clinical implications, there may be practical implications of the findings of the present study in as much as future standardised methodological guidelines for the assessment of L-FMC, as are available for FMD (Corretti et al. 2002; Thijssen et al. 2011), should consider recommending the avoidance of exercise in the period preceding L-FMC assessment.

In summary, the present study shows that acute dynamic exercise with a large muscle mass augments the radial artery L-FMC response in young healthy individuals. However, we did not observe an increase in the FMD response, but the composite end point (i.e., L-FMC + FMD) was augmented secondary to the greater L-FMC response. Future studies are required to investigate the time course of the effect of acute exercise on the L-FMC response and to determine the mechanisms underlying its attenuation immediately after dynamic exercise.

## Abbreviations

ANOVA: Analysis of variance
BP: Blood pressure
FMD: Flow-mediated vasodilatation
HR: Heart rate
L-FMC: Low-flow mediated constriction
MAP: Mean arterial pressure
RPE: Rating of perceived exertion
SR_AUC_: Shear rate area under the curve
